# Spatial analysis of urine zinc (Zn) concentration for women of reproductive age and school age children in Malawi

**DOI:** 10.1007/s10653-020-00700-5

**Published:** 2020-08-30

**Authors:** Felix P. Phiri, E. Louise Ander, R. Murray Lark, Edward J. M. Joy, Alexander A. Kalimbira, Parminder S. Suchdev, Jellita Gondwe, Elliott M. Hamilton, Michael J. Watts, Martin R. Broadley

**Affiliations:** 1 School of Biosciences, University of Nottingham, Sutton Bonington Campus, Loughborough, Leicestershire, LE12 5RD UK; 2 Department of Nutrition, HIV and AIDS, Ministry of Health, P. Bag B401, Lilongwe, Malawi; 3 Inorganic Geochemistry, Centre for Environmental Geochemistry, British Geological Survey, Nottingham, NG12 5GG UK; 4 Faculty of Epidemiology and Population Health, London School of Hygiene and Tropical Medicine, Keppel Street, London, WC1E 7HT UK; 5 Department of Human Nutrition and Health, Faculty of Food and Human Sciences, Bunda Campus, Lilongwe University of Agriculture and Natural Resources, P.O. Box 219, Lilongwe, Malawi; 6 Department of Pediatrics and Hubert Department of Global Health, Emory University, Atlanta, GA 30322 USA; 7 Community Health Sciences Unit, Ministry of Health, Private Bag 65, Lilongwe, Malawi

**Keywords:** Biomarker, Casual urine, Micronutrient survey, Sub-Saharan Africa, Urine zinc

## Abstract

Zinc (Zn) is an essential micronutrient, and Zn deficiency remains a major global public health challenge. Recognised biomarkers of population Zn status include blood plasma or serum Zn concentration and proxy data such as dietary Zn intake and prevalence of stunting. Urine Zn concentration is rarely used to assess population Zn status. This study assessed the value of urine Zn concentration as a biomarker of population Zn status using a nationally representative sample of non-pregnant women of reproductive age (WRA) and school-aged children (SAC) in Malawi. Spot (casual) urine samples were collected from 741 WRA and 665 SAC. Urine Zn concentration was measured by inductively coupled plasma mass spectrometry with specific gravity adjustment for hydration status. Data were analysed using a linear mixed model with a spatially correlated random effect for between-cluster variation. The effect of time of sample collection (morning or afternoon), and gender (for SAC), on urine Zn concentration were examined. There was spatial dependence in urine Zn concentration between clusters among SAC but not WRA, which indicates that food system or environmental factors can influence urine Zn concentration. Mapping urine Zn concentration could potentially identify areas where the prevalence of Zn deficiency is greater and thus where further sampling or interventions might be targeted. There was no evidence for differences in urine Zn concentration between gender (*P* = 0.69) or time of sample collection (*P* = 0.85) in SAC. Urine Zn concentration was greater in afternoon samples for WRA (*P* = 0.003). Relationships between urine Zn concentration, serum Zn concentration, dietary Zn intake, and potential food systems covariates warrant further study.

**Electronic supplementary material:**

The online version of this article (10.1007/s10653-020-00700-5) contains supplementary material, which is available to authorized users.

## Introduction

Zinc (Zn) is an essential micronutrient and is required for enzyme function, the metabolism of DNA and RNA, protein synthesis, and cell growth and differentiation (Lowe et al. 2009). It is one of the most abundant trace elements in the human body after iron and is involved in numerous metabolic processes (King et al. 2016). Zinc deficiency affects growth and development, with a range of health consequences (Hotz and Brown 2004). Zinc deficiency increases the risk of diarrhoea in children under 5 years old and is associated with high morbidity and mortality among mothers and new born infants (Wessells and Brown 2012). Globally, Zn deficiency is more prevalent in developing countries, especially in sub-Saharan Africa (SSA) where it is estimated at > 30% (World Health Organization 1996; Wessells and Brown 2012).

Three methods for assessing risk of Zn deficiency at population levels include analysis of plasma or serum Zn concentration as a biomarker, which can be compared to thresholds for different demographic groups, and proxy measures which include dietary Zn intake, and stunting (low height for age) (King et al. 2016; Walker and Black 2007; Wessells and Brown 2012). The use of plasma or serum Zn concentration has been endorsed by several committees (Hotz and Brown 2004; De Benoist et al. 2007). However, plasma or serum Zn concentration has several limitations, including being under homeostatic control across a wide range of Zn intakes (King et al. 2000). There is also considerable intra-individual variation, including being affected by fasting status which result in higher concentration than non-fasting, pregnancy, and influence of inflammation (Diana et al. 2017; Likoswe et al. 2020). Stunting is associated with many other factors including multiple nutrient deficiencies and access to animal source foods (King et al. 2016), and other dietary quality aspects including aflatoxin contamination of foods (Hoffmann et al. 2018).

In the most recent nationally representative Micronutrient Survey (MNS), Zn deficiency was reported of among > 60% of the Malawi population across demographic groups, based on standard thresholds of serum Zn concentration (National Statistical Office et al. 2017; Likoswe et al. 2020). These data are consistent with a high prevalence of stunting in Malawi (National Statistical Office and ICF 2017). Using household expenditure data, Joy et al. (2015b) estimated that 57% of households were not consuming enough Zn to meet the sum of member Estimated Average Requirements (EARs).

Urine Zn concentration is not an established biomarker of Zn status (King et al. 2016). However, decreased excretion of Zn in urine has been associated with Zn deficiency (Nishi et al. 1980) and urine Zn concentration has been used to diagnose Zn deficiency in apparently healthy individuals (Baer and King 1984; Gibson 1990). In a review by Lowe et al. (2009), urine Zn concentration was reported to be potentially useful as a biomarker, especially in persons with moderate Zn status which responded to changes in Zn status for all groups.

Excretion of Zn in urine varies diurnally due to differences in hydration status as reported for other elements such as selenium, iodine, arsenic, lead, and cadmium (Middleton et al. 2016b; Nermell et al. 2008; Phiri et al. 2020). Therefore, 24-h collection of urine would, in principle, be preferred compared with spot (casual) urine collection for clinical diagnosis of Zn deficiency (Gibson 1990; Pluhator et al. 1996). However, in large cross-sectional surveys, it is impractical to collect 24-h urine samples from large numbers of individuals. Urine Zn concentration also varies due to age and gender. Normalizing urinary biomarker concentrations is therefore critical across different demographic populations. To our knowledge, there are no studies that reported the variation of spot urine Zn concentration at national scale in SSA.

The purpose of this study was to assess the variation in spot urine Zn concentration among non-pregnant women of reproductive age (WRA) and school-aged children (SAC) using samples collected during Malawi’s 2015–2016 MNS. These demographic groups were used because urine was not collected from men and pre-school children (PSC) during the MNS. The specific questions addressed were:

1. Is there evidence of spatially dependent variation of spot urine Zn concentration among WRA and SAC in Malawi, as reported previously for urine Se concentration (Phiri et al. 2020)?
2. Is there variation in spot urine Zn concentration due to gender among SAC?
3. Is spot urine Zn concentration affected by time of sample collection (morning vs afternoon) among WRA and SAC?

## Methods

### Sampling

Background information on the 2015–2016 Malawi Demographic and Health Survey (MDHS) and MNS are documented fully elsewhere (National Statistical Office and ICF 2017; National Statistical Office et al. 2017; Phiri et al. 2019, 2020). The MDHS and MNS are periodic surveys which take place approximately every 5 years. Briefly, The MNS represented a subsample of the wider MDHS which was designed as a cross-sectional study, with a two-stage cluster sampling design to enable indicators to be obtained nationally in each of the 28 Districts of Malawi, for both urban and rural populations. The sampling frame was based on the Malawi Population and Housing Census of 2008. For the first stage of the MDHS, a total of 850 Enumeration Areas (EAs), also referred to as clusters, were selected from a total of more than 9000 EAs using a probability proportional to population size. For the second stage of the MDHS, 30 and 33 households were selected at random from each urban and rural cluster, respectively, using an updated list of the eligible 27,531 households within the 850 MDHS clusters. For the MNS, 105 target clusters were selected randomly from the MDHS clusters, stratified as 35 in each region of North, Central and South, so that 8 urban and 27 rural clusters were selected from each region. From each of the urban and rural MNS clusters, 10 and 11 households, respectively, were excluded from the MNS because these households had been selected for HIV testing within the MDHS. Therefore, the MNS comprised 20 and 22 households from each of the urban and rural clusters, respectively, giving a target sample size of 480 and 1782 urban and rural households, respectively.

Exclusion criteria were applied to participants based on the requirements of the MNS and not for the purposes of this study. Ethical approval and a Material Transfer Agreement (MTA) for sample shipping and analyses were granted by the Malawi National Health Sciences Research Committee (NHSRC), reference number NHSRC 15/5/1436. Individual informed consent for WRA and assent for SAC and their caregivers was secured by the MNS field team prior to sample collection. Samples were handled in the UK under the terms of a Human Tissue Authority (HTA) licence.

### Urine sample collection

Spot urine specimens were collected from households selected for the 2015–2016 MNS, to test for urine iodine concentration and other health indicators. Samples were collected from non-pregnant WRA (age 15–49 years) and SAC (age 6–14 years, female and male), but not from men or pre-school children (PSC). A spot urine was collected at the time of interview or within 24 h if that was not possible. No other information on the urine samples (e.g. flow rate, time since last void) was collected.

Urine sample collection was undertaken as described by Phiri et al. (2020). Strict quality control measures were followed in sample management using well-trained nurses, clinicians, and laboratory technicians under the supervision of the Centers for Disease Control and Disease Prevention (CDC), Georgia, USA. Samples were collected in 125-mL polypropylene urine cups with screw caps (model 1131202, Heinz Herenz Medizinalbedarf GmbH, Hamburg, Germany). In total, 1406 urine samples were collected from WRA (*n* = 741) and SAC (*n* = 665). Cold chain management was followed as described previously (Phiri et al. 2020). Samples were transferred frozen to the UK for analysis, and residual samples were disposed of in accordance with the MTA and HTA licence following the sample analyses.

### Analysis of zinc concentration in urine

Total elemental concentrations of a routine suite of approximately 30 elements were determined in thawed urine samples following a × 10 dilution in 1% v/v HNO_3_, 0.5% v/v HCl using an Agilent 7500cx series inductively coupled plasma mass spectrometer (ICP-MS, Agilent Technologies, Santa Clara, USA) under the conditions described in Middleton et al. (2016a, b). Zinc was measured in helium (He) reaction mode to reduce polyatomic interferences associated with ^66^Zn, such as ^34^S^16^O_2_^+^. Internal standardization was achieved through the simultaneous introduction of germanium (Ge) via a T-piece. The limit of detection for Zn was 19 µg L^−1^ based on 3 × standard deviation of 137 analytical blanks. Certified reference materials (CRMs) were analysed with urine samples: NIES No. 18 Human Urine (National Institute for Environmental Studies, Tsukuba, Japan) (total Zn certified value: 620 ± 50 µg L^−1^; recovery: 103%; precision: 6%; *n* = 57) and Seronorm Trace Elements Urine L-1 (LGC Group, Teddington, UK) (total Zn certified value: 334 µg L^−1^, recovery: 102%; precision: 10%; *n* = 16).

### Specific gravity adjustment for hydration status

We report urine Zn concentration data adjusted for hydration status using the specific gravity (sg) method, as in Phiri et al. (2020) where sg was a suitable adjustment method for urine Se concentration for both WRA and SAC. Other studies have also used sg to adjust urine Zn concentration (Moore et al. 2017, 2019; Middleton et al. 2019). The sg was measured using a handheld temperature-corrected refractometer (PAL-10S, Atago, Japan), on 1 to 2 drops of urine. For any given sample, the measured Zn concentration, Zn_uncor_, was specific gravity adjusted to give a value Zn_sg_ as follows:

1 $$ {\text{Zn}}_{{{\text{sg}}}} = {\text{Zn}}_{{{\text{uncor}}}} \times \frac{{\left( {{\text{sg}}_{{{\text{Mean}}}} - 1} \right)}}{{({\text{sg}}_{{{\text{Meas}}}} - 1)}} $$

where $\mathrm{sg}$_Mean_ is the mean of specific gravity of all samples tested in the study, ${\mathrm{sg}}_{\mathrm{Meas}}$ refers to specific gravity measured on the individual sample. Methods followed those in Levine and Fahy (1945) and Middleton et al. (2016a, b), and all urine Zn concentration data are reported on this basis.

### Data analysis

The main objective of the analysis was to examine the spatial variation in urine Zn concentration from SAC and WRA. In addition, we examined whether there were gender differences in urine Zn concentration among SAC, and whether urine Zn concentration varied between morning and afternoon sampling times for both SAC and WRA given the evidence that plasma and serum Zn concentration is greater in the afternoon (King et al. 2016).

### Exploratory analyses

Inspection of exploratory statistics and histograms indicated that urine Zn concentration data for both WRA and SAC were best analysed after transformation to natural logarithms (log_e_) so that the assumption of normally distributed variation was plausible. We assessed the evidence for spatial correlation in the between-cluster variance component by comparing two models for it, one with a Matérn spatial correlation function (Stein 1999) and one in which the between-cluster component of the model was assumed to be independent. Both those modes had a constant mean as the only fixed effect. These two models were compared on the Akaike information criterion (AIC). Where evidence was found for the more complex model with spatial correlation, we then went on map urine Zn concentration by computing the best unbiased linear prediction at nodes on a fine grid (see Supplementary Material), which is the ordinary kriging predictor in this case, so that these could be visualized. We assessed the weight of evidence against the null hypotheses of no gender difference and no time of sampling difference with the Wald statistics.

### Linear mixed model

A linear mixed model (LMM) was used, which reflects the hierarchical sampling design (individuals selected within households and households within clusters). This is the random effects structure; the fixed effects were either (1) just a constant mean or (2) gender (SAC only) or (3) time of sampling (morning = AM vs afternoon = PM) for both groups. We assessed the weight of evidence against the null hypotheses of no gender difference and no time of sampling difference with the Wald statistics. We assessed the evidence for spatial correlation in the between-cluster variance component by comparing two models for it, one with a Matérn spatial correlation function (Stein 1999) and one in which the between-cluster component of the model was assumed to be independent. Both those models had a constant mean as the only fixed effect. These two models were compared on the AIC. Where evidence was found for the more complex model using spatial correlation, we then went on to apply ordinary kriging to map urine Zn concentration so that these could be visualized. For the final analysis, we used an LMM, which took the form:

2 $$ {\mathbf{y}} = {\mathbf{X\tau }} + {\mathbf{Z}}_{C} {{\varvec{\upeta}}} + {\mathbf{Z}}_{{\text{H}}} {{\varvec{\upalpha}}} + {{\varvec{\upvarepsilon}}} $$

where **y** is a vector of *n* observations of log_e_-transformed urine Zn concentration, **X** is a design matrix with *n* rows and a column of 1s (i.e. the value 1 in each cell of that column of the matrix) as it corresponds to a constant fixed effect, either the overall mean (for the null model) or the intercept in a more complex fixed effects model. For models where gender or time was included in the fixed effects, a second column was included in **X** with value 0 in elements corresponding to the reference level of the effects (i.e. “male” for gender or “AM” for time) and 1 for the second level (“female” or “PM”). The term **τ** is a vector containing the fixed effects parameters, either the estimated mean, when this is the only fixed effect, or the estimated mean for the reference level of the fixed effect and the difference between the means for the second level and reference level. The term **η** is a random component, with a random value for the mean difference between the fitted and observed transformed urine Zn concentration under the model in each cluster of the original sample design. Each entry in this random vector is associated with the corresponding observation by the design matrix **Z**_C_ which indicates the cluster to which each observation belongs. Similarly, the term **α** is a random effect which represents the deviation between the mean error of the fixed model within each household and the mean for the cluster to which that household belongs. The term **Z**_H_ is the design matrix which associates each observation with its corresponding household. Finally, **ε** is a random effect, the random deviation of the observation for each individual from its corresponding household mean.

In the LMM, Eq. (2), the two random effects, **ε** and **α** are independent Gaussian random variables, each identically and independently distributed with mean zero. The variances of these random effects are of interest, namely the variance of **ε** (the between-individual within household variance) and the variance of **α** (the between-household within cluster variance). The random effect **η**, the between-cluster random component, is modelled as a spatially correlated random variable so the observations have a zero mean and a covariance matrix which takes the form:

3 $$ {\mathbf{V}} = \sigma_{{\text{C}}}^{{2}} {\mathbf{R}}, $$

where *σ*_C_^2^ is the between-cluster variance and the correlation matrix **R** has entries:

4 $$ {\mathbf{R}}\left[ {{\text{i}},{\text{j}}} \right] = \rho \left( {\left| {{\mathbf{x}}_{{\text{i}}} {-}{\mathbf{x}}_{{\text{j}}} } \right|;\kappa ,\phi } \right), $$

where **|x**_**i **_− **x**_**j**_**|** denotes the distance in space between the locations, **x**_**i**_ and **x**_**j**_, of the **i**th and **j**th cluster, respectively, and *ρ*(*u*; *κ*,*ϕ*) is a Matérn correlation function (Matérn 1986) where *u* is the lag distance between two locations in space and *κ* and *ϕ* are parameters which determine, respectively, the spatial smoothness of the variation of **η** over short distances and the rate at which the autocorrelation of the variable decays with distance in space. In this study, with coordinates on a UTM projection, distances in space were Euclidean distances on the plane.

Stein (1999) provides details of the Matérn correlation function. Briefly, the parameter *κ* controls the smoothness of the spatial random variable whereas *ϕ* controls, with *κ*, the spatial scale over which the variable shows spatial dependence (Stein 1999). The larger the value of *κ* the smoother is the spatial variation of the random variable. The overall LMM therefore has five parameters for the random variation, the two parameters of the Matérn correlation function and the variances of the three random effects. These were estimated by residual maximum likelihood (REML) (Diggle and Ribeiro 2007) using code written for the R platform (R Core Team 2017). In normal maximum likelihood, it is well-known that estimates of variance parameters are biased because they depend on estimates of the fixed effect parameters (in **τ**). The use of REML minimizes this bias by computing a likelihood which depends on generalized increments of the observations, which filter out the fixed effects. Once the variance parameters are estimated, they can be used to estimate the fixed effects parameters by weighted least squares (Lark and Cullis 2004). One can also compute the standard error of these estimates. More information on the REML method is provided as supplementary material. We followed Diggle and Ribeiro (2007) in estimating the *κ* parameter by a profile likelihood approach, considering some discrete values of *κ* (0.1, 0.5, 1 and 2), fixing *κ* at each of these values in turn and finding the corresponding REML estimates of the remaining parameters (Diggle and Ribeiro 2007). The model for which the likelihood was largest was then selected. To characterize the scale of spatial dependence of a fitted model, we computed the effective range. This is the lag distance at which the Matérn correlation takes a small value (0.05) and can be obtained by multiplying the parameter *ϕ* by a constant which depends on the value of *κ*. In this case, with *κ* = 0.5, the effective range was 3 times *ϕ*.

This random effect structure was used for all models, i.e. for a constant mean as the only fixed effects, and for models with, respectively, gender (SAC only) and sample time (AM or PM) as fixed effects. In addition, a second model was fitted with a constant mean as the only fixed effect but with no spatial dependence assumed for the random term **η**. This was done to assess the evidence for spatial dependence prior to spatial mapping. The two models were compared on the AIC, (Akaike 1973) which is computed as:

5 $$ A = 2P - 2l, $$

where *P* is the number of model parameters and *l* is the negative log_e_-likelihood. A rule to select the model for which *A* is smallest accounts for likelihood while penalising model complexity. The rule minimizes the expected value of a statistical measure of the divergence between the estimated model and the process that generates the data (Buckland et al. 1997). In our case, we examine AIC for the model with spatial dependence for the between-cluster random effect, and for the alternative with no spatial dependence, to assess the evidence for spatial structure of this random component of the model.

The evidence for the effect of gender or of sample time was tested by computing a Wald statistic. Recall that the model was set up such that, under a null hypothesis of no difference, the value of **τ**[2], the second element of **τ** in Eq. (2), would be zero. The Wald statistic is computed as the square of the estimate of **τ**[2] to its standard error and, under the null hypothesis, it has a chi-squared distribution with 1 degree of freedom. A nonzero value of *W* is evidence against the null hypothesis, and a *P* value to assess that evidence is easily obtained.

### Mapping urine zinc concentration in SAC

Spatial prediction of urine Zn concentration was based on the LMM with a constant mean as the only fixed effect. We proceeded to spatial prediction only if the AIC indicated that there was evidence for spatial correlation in the between-cluster random effect. In this case, an ordinary kriging estimate of individual urine Zn concentration on a log_e_ scale was computed at nodes of a 500 by 500 m square grid. These estimates, $\stackrel{\sim }{y}$, were transformed back to the original scale of measurement by exponentiation, $\stackrel{\sim }{z}= {e}^{\stackrel{\sim }{y}}$, which gives a median-unbiased central value of the prediction distribution on the original scale (Pawlowsky-Glahn and Olea 2004). These predictions were then visualized using ArcGIS (v10.3, ESRI, Redlands, CA, USA).

## Results

### Urine zinc concentration

In both WRA and SAC groups, the mean was greater than median (Table 1), which is consistent with the large positive coefficients of skewness (larger than 1) and octile skewness (larger than 0.2).

**Table 1:** Urine Zn_sg_ concentration (µg L^−1^) for women of reproductive age (WRA) and school age children (SAC) in Malawi

Variable	Mean	Median	SD	min	max	S	OS
WRA (*n* = 741)							
Zn_sg_	410.2	321.6	495.5	9.0	11,667.7	15.2	0.3
log_e_ Zn_sg_	5.7	5.8	0.8	2.2	9.4	− 0.5	− 0.1
SAC (*n* = 665)							
Zn_sg_	412.2	345.9	305.8	30.0	2943.9	2.3	0.3
log_e_ Zn_sg_	5.8	5.8	0.7	3.4	8.0	− 0.4	− 0.1

*n* = sample number in the demographic group

*SD* standard deviation, *S* skewness value, *OS* octile skewness value, *log*_*e*_ natural logarithm, *min* minimum, *max* maximum

### Linear mixed model parameters for urine zinc concentrations

Table 2 shows the components of variance of urine Zn concentration (log_e_ scale) for WRA and SAC in a model for variation about the overall mean value of the population. The results show that, in both WRA and SAC, the between-individual variance was the dominant component and the between-cluster variance component was an order of magnitude smaller. The between-household variance component was small (comparable to the between-cluster component) for WRA but was of the same order of magnitude as the between-individual component for SAC.

**Table 2:** Variance of random effects for urine Zn concentration in women of reproductive age (WRA) and school age children (SAC) in Malawi

Random model	Fixed model	Variance between-individuals within HH	Variance between-HH within clusters	Variance between clusters	*ϕ* for between-cluster random effects	Effective range	AIC	Wald test	*P* value for Wald test
WRA									
Spatial dependence	Constant mean	0.500	0.048	0.079	37.3	111.9	391.2		
Spatial dependence	Sampling time	0.498	0.043	0.076	32.5	97.6		8.81	0.003
No spatial dependence	Constant mean	0.512	0.026	0.080			389.2		
SAC									
Spatial dependence	Constant mean	0.331	0.159	0.068	74.7	224.1	202.6		
Spatial dependence	Sampling time	0.331	0.161	0.068	74.4	223.2		0.04	0.85
Spatial dependence	Gender	0.332	0.159	0.067	75.2	225.5		0.16	0.69
No spatial dependence	Constant mean	0.332	0.149	0.064			207.0		

The Wald test assesses the evidence against a null hypothesis of equal means for log_e_ urine Zn concentration for either morning (AM) and afternoon (PM) sample times (WRA and SAC) or for male and female participants (SAC). In these instances, it is distributed as *χ*^2^ with one degree of freedom. The Matérn correlation parameters *κ* and *ϕ* are described in the text where they appear in Eq. 4. Note, *κ* was set to 0.5 after examination of profile likelihoods, and the random variation is spatially smoother with constant *κ* (0.5). The *κ* parameter was selected based on maximum profile likelihood, following the procedure described by Diggle and Ribeiro (2007). The *κ* parameter selected indicates that the random variation in urine Zn concentration is spatially smoother in WRA than in SAC

*HH* household, *AIC* Akaike information criterion

**Fig. 1:**
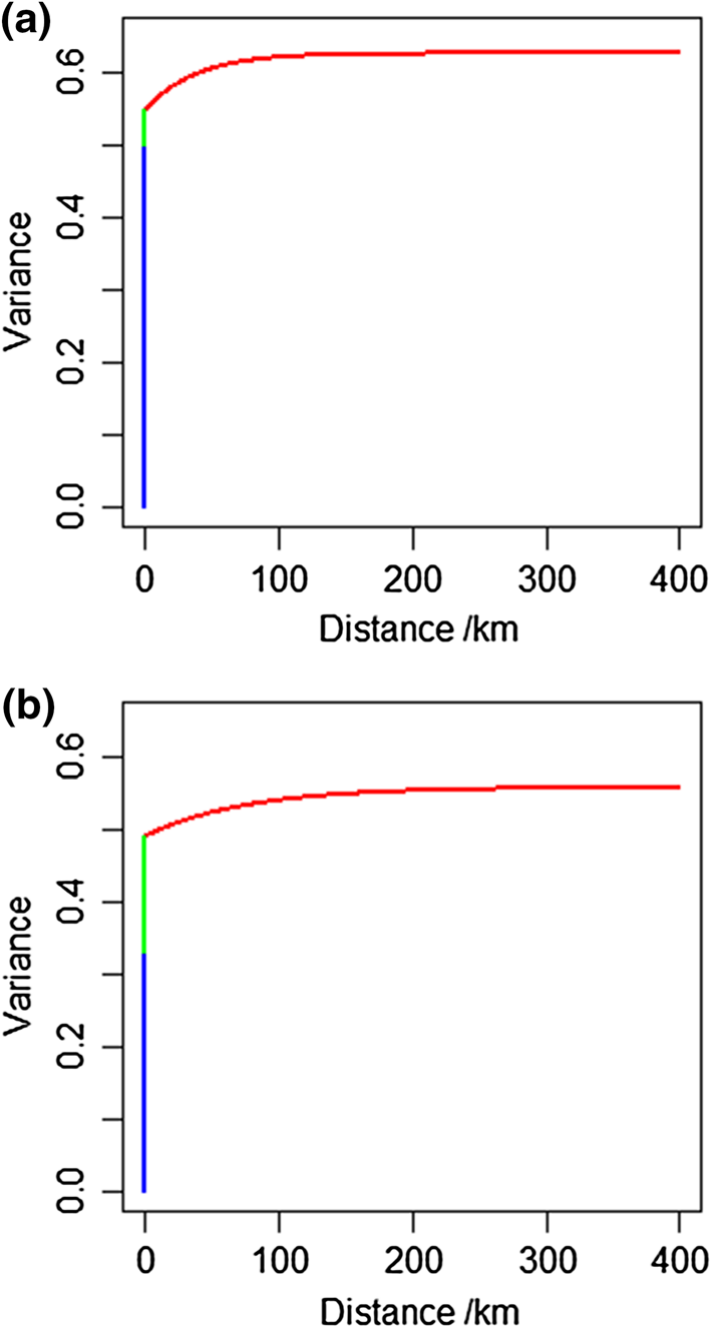
Random variation of urine Zn concentration based on variograms for **a** women of reproductive age (WRA) and **b** school age children (SAC) in Malawi. The blue line is the variance component from between-individual within household, the green line between-household within-cluster, and the red line between-cluster showing spatial dependence. The Akaike information criterion (AIC) in Table 2 is the measure of evidence for the spatial dependence of this component

For both WRA and SAC, the spatial (between-cluster) component of variance was small relative to the between-individual variance. In Fig. 1a, b, the random effects model is shown in the form of variograms (half the expected square difference between two urine Zn concentration measurements (log_e_ scale) as a function of distance in space between them). Note that these variogram plots are derived directly from the variance parameter estimates for the linear mixed model, as a convenient way to visualize the spatial structure that these parameters described. The between-individual and between-household variance components contribute to the “nugget” component of these plots (their apparent intercept), and the between-cluster component depends on distance, given the *κ* and *ϕ* parameters for which estimates are given in Table 2. The variogram increases with distance up to an “effective range” of spatial dependence. In the case of WRA, the estimated effective range of spatial dependence (approximately 100 km) was shorter than for SAC (approximately 200 km). In fact, when we examine the values of AIC in Table 2, it was apparent that the evidence for spatial dependence in the between-cluster variation for WRA was weak. (AIC was larger for the model with spatial dependence.) We have no reason to regard the between-cluster variation for WRA as anything other than independent random variation, and so there was no basis for spatial mapping of this variable.

In contrast, for SAC, the value of AIC was smaller for the model with spatial dependence. On this basis, it was possible to make spatial predictions of urine Zn concentration from the data and the model by ordinary kriging (Diggle and Ribeiro 2007), in which observed values are linearly combined to make a prediction at an unsampled site. The ordinary kriging prediction is based on the assumption that the variable has an unknown but constant mean. The expected squared prediction error (kriging variance) is minimized by the ordinary kriging predictor, given this assumption and conditional on the estimated random effects parameters of the linear mixed model. Ordinary kriging predictions on a fine grid are presented as map in Fig. 5.

### Differences between genders in urine zinc concentration

**Fig. 2:**
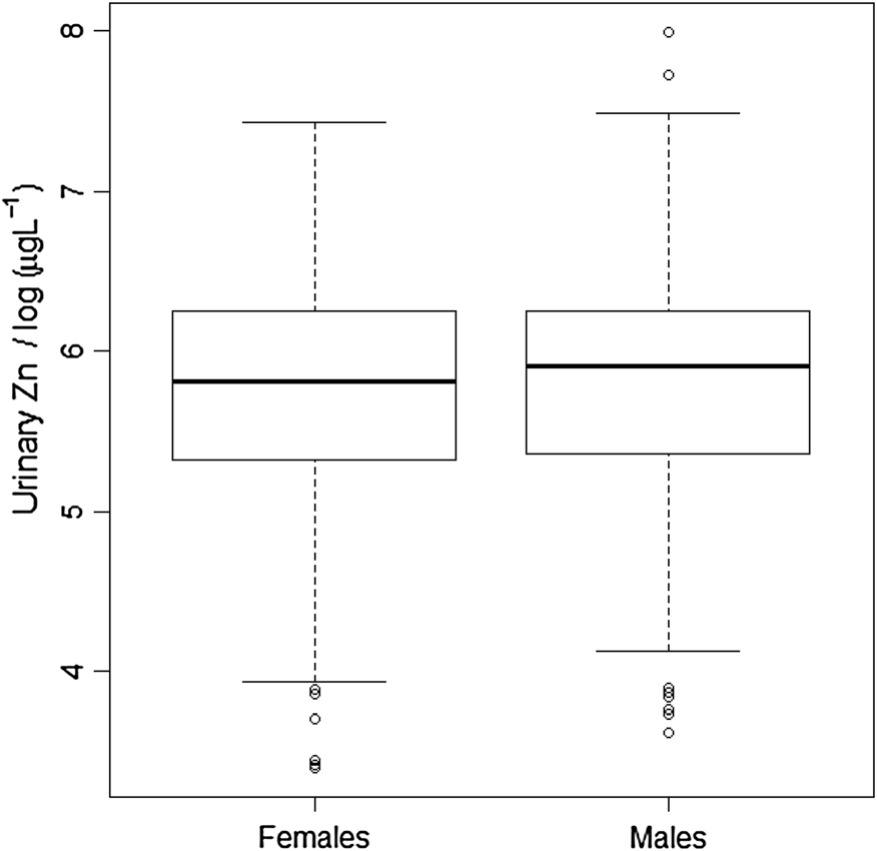
Urine Zn concentration (transformed to natural logarithms) of school age children (SAC) in Malawi, grouped according to gender

The Wald statistic for the null hypothesis that mean urine Zn concentration does not differ between SAC of different gender is close to zero (0.16) and provides no evidence to reject this null hypothesis (Table 2). Similarly, the box plots (Fig. 2) show no significant difference in urine Zn concentration by gender.

### Effect of time of collection (morning “AM” vs afternoon “PM”)

**Fig. 3:**
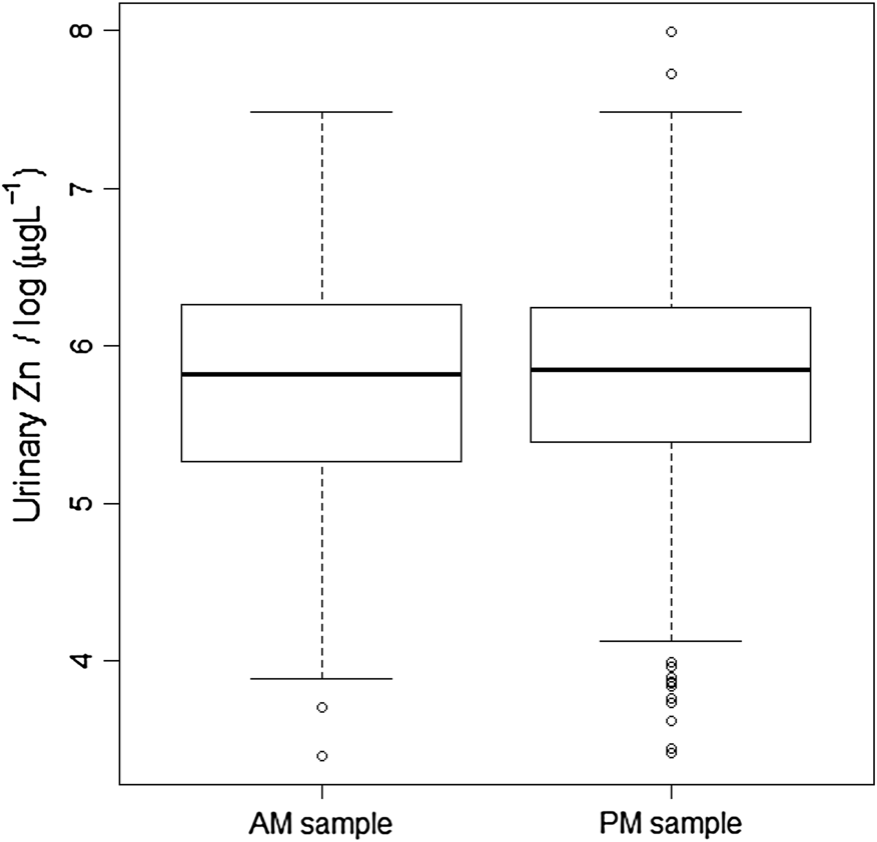
Urine Zn concentration (transformed to natural logarithms) of school age children (SAC) in Malawi, grouped according to whether the sample was taken in the morning (AM) or afternoon (PM)

**Fig. 4:**
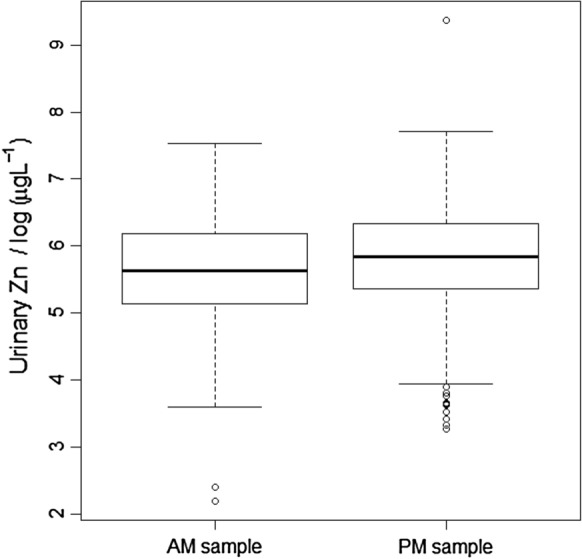
Urine Zn concentration (transformed to natural logarithms) of women of reproductive age (WRA) in Malawi, grouped according to whether the sample was taken in the morning (AM) or afternoon (PM)

The Wald statistic for the null hypothesis that the mean urine Zn concentration in morning and afternoon samples was the same was close to zero for SAC (0.04) providing no evidence to reject the null hypothesis. This was supported by Fig. 3, which shows the mean urine Zn concentration was only slightly larger for the afternoon samples (the difference is 0.013 log_e_(µg L^–1^) on the transformed scale, equivalent to a factor of just 1.4%). By contrast, for WRA the Wald statistic was larger (8.81) providing evidence to reject the null hypothesis (*P* = 0.003). This is supported by Fig. 4; urine Zn concentration was 0.172 log_e_(µg L^–1^) larger in the afternoon than in the morning sample on the transformed scale, equivalent to a factor of 19%.

## Discussion

The present study explored whether urine Zn concentration is a useful biomarker of Zn status which might contribute to evidence on the Zn status of the Malawi population. One challenge with assessing the value of urine Zn concentration as a biomarker is that there is no definitive standard biomarker against which to compare it. Although plasma or serum Zn concentration is often used in population assessments, its concentration is under tight homeostatic control with only ~ 1% of total body Zn found circulating in blood and this varies according to demographic group and inflammation status (King et al. 2016; Likoswe et al. 2020). A second challenge is that it is difficult to compare urine Zn concentration between studies of different population sampling frames and varying adjustments for hydration. Data on serum Zn concentration from the MNS show that 62.5% of WRA in the Malawian population were below the deficiency thresholds of 66 µg dL^−1^ for morning samples and 59 µg dL^−1^ for afternoon samples; 60.2% of SAC were below specific thresholds set for different age, gender, and sampling times (National Statistical Office et al. 2017). Low dietary supplies of Zn have been reported among the general Malawian population, with inadequate dietary Zn supplies among an estimated 57% of households (Joy et al. 2015b).

Using comparable analytical techniques and specific gravity adjustments, the median urine Zn concentration for WRA (321.6 µg L^−1^) in Malawi was less than observed for adult populations in Kenya (*n* = 55; median 479 µg L^−1^) and Tanzania (*n* = 190; 427 µg L^−1^) (Middleton et al. 2019). The studies conducted in Kenya and Tanzania were targeted to specific subnational areas and were not intended to be a nationally representative population. In Canada, urine Zn concentration has been reported as 350 µg L^−1^ (*n* = 2980; Health Canada 2013) and in Tarragona Province in Spain as 698.7 µg g^−1^ (Schuhmacher et al. 1994) among adults. Surprisingly, the Malawi urine Zn concentrations are higher than medians for Belgian and UK adult populations, i.e. 256 µg L^−1^ (*n* = 1001; (Hoet et al. 2013) and 180 µg L^−1^ (*n* = 132; Morton et al. 2014), respectively. However, these comparisons should not be over-interpreted given sampling and methodological differences; for example, these Belgian and UK values are based on data which are unadjusted for hydration status.

### Mapping urine zinc concentration for SAC

**Fig. 5:**
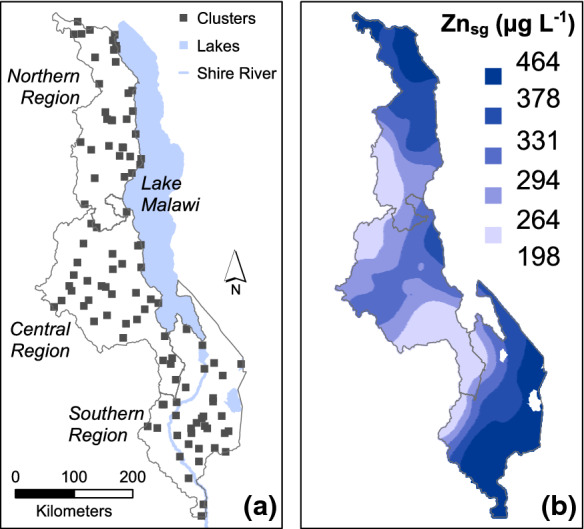
**a** Enumeration area (EA) locations and **b** predicted urine Zn_sg_ concentration across Malawi for school age children (SAC), adjusted using specific gravity

The broad spatial pattern of urine Zn concentration in SAC is shown in Fig. 5. Urine Zn concentrations are greater in parts of the north and south of Malawi (Northern Region and Lower Shire Valley) compared to the larger part of central-west, south-west, and north-west. A contributing factor to these differences might be due to soil type. For example, there is some evidence that maize grain Zn concentration is greater on some soil types in Malawi (Chilimba et al. 2011; Joy et al. 2015a, b). Local food system characteristics, including cropping patterns, may also influence urine Zn concentration. For example, legume production and consumption is likely to be greater in areas in which larger urine Zn concentrations are mapped, and legumes typically have larger grain Zn concentrations than maize (Joy et al. 2015b, 2017). Fish consumption is also likely to affect Zn intake and status. Further work is needed to understand whether the spatial difference in urine Zn concentration is due to geographical differences in soil type and dietary patterns. Further work is also needed to understand why the same degree of spatial dependency was not seen in WRA as compared with SAC.

### Influence of gender (SAC) and the time of sample collection in urine zinc concentration in school aged children and women of reproductive age group

There was no evidence of consistent differences in urine Zn concentration by gender in SAC (Fig. 2), consistent with serum Zn concentration in the same group which reported no difference due to gender (National Statistical Office et al. 2017). This could be because both males and females in this age group in Malawi have similar access to foods at home and through school feeding programmes and similar physical activity level. Similarly, the time for collection of samples (morning vs afternoon) in SAC had no effect on urine Zn concentration (Fig. 3). This contrasts with reported studies on plasma Zn concentration which reported effects of time, sex, and gender (King et al. 2016). Contrary to SAC, among WRA, urine Zn concentration was greater when samples were collected in the afternoon. This could be attributed to metabolic or physiological conditions, and strenuous physical exercise, which can increase urine Zn excretion (King et al. 2016; Zlotkin 1989). Thus, it is possible that the physical activity of WRA increases in the afternoon, for example, walking to collect firewood and other activities, although more detailed studies would be required to explore such relationships.

## Conclusions

Spot urine may be a cost-effective and less-invasive method for assessing Zn status among WRA and SAC, compared to serum or plasma Zn concentrations. Spatial analysis for urinary Zn status can be undertaken in SAC; however, more evidence needs to be generated as to why spatial structure exists for SAC but not WRA. Further work is needed to determine whether the measurement of urine Zn concentration in spot urine samples could be integrated as part of the ongoing nutrition surveillance systems, such as the MNS and other routine systems which are already collecting spot urine for population iodine monitoring. Given the variability in urine Zn concentration due to time of sample collection in WRA, an appropriate survey design would be needed to deduce environmental factors that may influence urine Zn concentration. There is also a need to establish the sensitivity, specificity, and threshold values of urine Zn concentration for indicating Zn deficiency, including whether urine Zn concentration is responsive to changes in Zn intake.

## Electronic supplementary material

Below is the link to the electronic supplementary material.

Supplementary file1 (DOCX 53 kb)
