# Supplementary material for: Spatial analysis of urine zinc (Zn) concentration for women of reproductive age and school age children in Malawi
Source: Environ Geochem Health. 2020 Aug 30;43(1):259–71. doi: 10.1007/s10653-020-00700-5 (PMC7847879; doi:10.1007/s10653-020-00700-5)
Supplement: Supplementary file 1 — Supplementary file1 (DOCX 53 kb) [file 10653_2020_700_MOESM1_ESM.docx]

**The Linear Mixed Model, Residual Maximum Likelihood and the Ordinary Kriging Predictor.**

The Linear Mixed Model (LMM) as used in the main paper is presented in Equation (2):

**y** = **W** + **Z**_C_**** + **Z**_H_**α**+ **ε** . (S1)

As explained in the text, the term **y** is a vector of *n* observations of log*_e_*-transformed urine Zn concentration and **W** is a design matrix which contains the fixed effects, in this case either a column vector of ones for a constant mean, or, as described in the main text, indicating the levels of other fixed effects (gender or time of sampling). The remaining terms are random effects, all of zero mean. The first of these is the between-cluster random effect, **Z**_c_, which has the covariance matrix **V** defined in Equation (3) of the main paper. The between-household within-cluster random effect **Z**_H_ has a *n*×*n* covariance matrix **C**_H_, which is defined as

**C**_H_ = σ^2^_H_**U**_H_**U**^T^_H_, (S2)

where **U**_H_ is a *n*×*n_k_* design matrix with all elements set to zero except for one element in each row which takes the value 1. If the element in the ***j***th column of the ***i***th row is 1 this indicates that the ***i***th observation belongs to the ***j***th household. The superscript T denotes a matrix transpose, and σ^2^_H_ is the between-household variance.

The between-individual within-household covariance matrix **C**_I_ is simply defined as

**C**_I_ = σ^2^_I_**I***_n_*, (S3)

where **I***_n_* is the *n*×*n* identity matrix and **σ^2^_I_** is the between-individual within-household variance.

An overall covariance matrix for **y** may then be written as

**C** = **V** + **C**_H_ + **C**_I_ . (S4)

The parameters required to characterize the LMM of our data are the two variance components in Equations (S2) and (S3) above, and the between cluster variance, **σ^2^_C_** and parameters of the correlation function (******* *) set out in Equation (4) of the main paper.We do not describe the residual maximum likelihood (REML) approach to estimation of these parameters in detail here, and refer the reader elsewhere (e.g. Lark and Cullis, 2004). In summary, the residual likelihood does not depend on the fixed effects parameters in **** but ratheron what are called generalized increments of the data, computed on the basis of the design matrix **W**. This is directly comparable to the process of taking successive differences between observations in a time series to filter out a non-stationary mean. The log*_e_* residual likelihood, given some proposed set of random effects coefficients from which **C** can be computed, is

*l*(σ^2^_C_,σ^2^_H_,σ^2^_I_,*  *;**y**)= −½ (ln |**C**| + ln |**W**^T^**C**^−1^**W**| + **y**^T^**Py**), (S5)

where |·| denotes the determinant of a matrix and the superscript ^−1^ denotes the inverse of a matrix, and **P** is defined as

**P** = **C**^−1^ − **C**^−1^**W**(**W**^T^**C**^−1^**W**)^−1^ **W**^T^**C**^−1^.

The optim procedure was used on the R platform (R Core Team, 2017) to find values of the parameters σ^2^_C_,σ^2^_H_,σ^2^_I_,* * which maximize the residual likelihood, given a fixed value of* * as described in the main paper, and, following Diggle and Ribeiro (2007), the value of * * was found for which the residual likelihood was largest. We used the default optimizer of optim, which is the simplex algorithm of Nelder and Mead (1965), setting the scaling factor for each variance parameter to the reciprocal of the absolute value of the partial derivative of the residual likelihood with respect to that parameter at an initial estimate.

Once the REML estimates of the variance parameters are obtained, then a corresponding covariance matrix **C*** can be specified for the observations, and the fixed effects coefficients can by estimated by

***** = (**W**^T^**C***^−1^**X**)^−1^ **W**^T^**C***^−1^**y.** (S6)

The covariance matrix of these estimates is given by

**H** = (**W**^T^**C***^−1^**W**)^−1^

From which their standard errors are obtained.

With the fixed and random effects estimated, it is possible to proceed to the prediction of values for unknown individuals, in this case an individual at an unsampled site. We assume that the mean value of transformed urine Zn concentration is an unknown constant over the sampled region, and our prediction at any location is therefore equivalent to the ordinary kriging prediction, with the variance components, σ^2^_H_ and σ^2^_I_, both treated as components of the uncorrelated “nugget” variance. The ordinary kriging prediction of transformed urine Zn concentration of an individual at a notional unsampled cluster at location **x**_0_ site is computed by

$$\tilde{Y}= \sum_{i=1}^{n} \lambda_{i}y_{i}$$

where λ_i_ is a weight attached to the *i*th observation, and where the weights are constrained to sum to one. This prediction is optimal in the sense that the expected squared error of the prediction from our *n* observations is minimized for a set of weights obtained by the ordinary kriging equation

**w** = **K**^−1^**k** (S7)

where

$\text{K }\text{= }\left[ \begin{matrix} \mathbf{C}^{\boldsymbol{*}} & \boldsymbol{1}_{n} \\ \boldsymbol{1}_{n} & 0 \end{matrix} \right]$,

$\text{k }\text{= }\left[ \mathbf{c},1 \right]^{T}$,

where c is the vector of covariances between the *n* observations and the unsampled individual at location **x**_0_, derived from the variance parameters of the LMM, and

$\text{w }\text{= }\left[ \lambda_{1},\lambda_{2},\ldots\lambda_{n},\mu\right]^{T}$,

where *μ* is a Lagrange multiplier.

The prediction error variance is

^^_OK_ = σ^2^_H_+σ^2^_H_+σ^2^_H_ – **k**^T^**w**. (S8).

**Additional Reference**

Nelder, J. A., and Mead, R. (1965). A simplex algorithm for function minimization. *Computer Journal*, *7*(4), 308–313.

**Fixed effect coefficient estimates for time of day and gender effects**

Note, we set up the design matrix for fixed effects using corner-point constraints, which means that the first coefficient is the mean for the reference level of a factor (AM sampling times, or Female SAC), and the second coefficient is the contrast between the mean for the second level of the factor (PM sampling time or Male SAC) and the reference level.

**AM**= Morning, **PM**=Afternoon, **SAC**=School aged children, **WRA**=Women of Reproductive age group
